# RETRACTION: Effect of Dexmedetomidine Combined With Local Infiltration Analgesia on Postoperative Wound Complications in Patients With Total Knee Arthroplasty: A Meta‐Analysis

**DOI:** 10.1111/iwj.70352

**Published:** 2025-03-17

## Abstract

**RETRACTION:** JiangC. , LvC. , GaoJ. , LiC. , “Effect of Dexmedetomidine Combined With Local Infiltration Analgesia on Postoperative Wound Complications in Patients With Total Knee Arthroplasty: A Meta‐Analysis,” International Wound Journal 21, no. 2 (2024): e14381, 10.1111/iwj.14381.PMC1082507337772317

The above article, published online on 29 September 2023, in Wiley Online Library (http://onlinelibrary.wiley.com/), has been retracted by agreement between the journal Editor in Chief, Professor Keith Harding; and John Wiley & Sons Ltd. Following an investigation by the publisher, all parties have concluded that this article was accepted solely on the basis of a compromised peer review process. The editors have therefore decided to retract the article. The authors did not respond to our notice regarding the retraction.

**RETRACTION:** C. Jiang , C. Lv , J. Gao , C. Li , “Effect of Dexmedetomidine Combined With Local Infiltration Analgesia on Postoperative Wound Complications in Patients With Total Knee Arthroplasty: A Meta‐Analysis,” International Wound Journal 21, no. 2 (2024): e14381, 10.1111/iwj.14381.PMC1082507337772317

The above article, published online on 29 September 2023, in Wiley Online Library (http://onlinelibrary.wiley.com/), has been retracted by agreement between the journal Editor in Chief, Professor Keith Harding; and John Wiley & Sons Ltd. Following an investigation by the publisher, all parties have concluded that this article was accepted solely on the basis of a compromised peer review process. The editors have therefore decided to retract the article. The authors did not respond to our notice regarding the retraction.

